# Serotypes, intimin variants and other virulence factors of *eae *positive *Escherichia coli *strains isolated from healthy cattle in Switzerland. Identification of a new intimin variant gene (eae-η2)

**DOI:** 10.1186/1471-2180-5-23

**Published:** 2005-05-09

**Authors:** Miguel Blanco, Sandra Schumacher, Taurai Tasara, Claudio Zweifel, Jesús E Blanco, Ghizlane Dahbi, Jorge Blanco, Roger Stephan

**Affiliations:** 1 Laboratorio de Referencia de *E. coli *(LREC), Departamento de Microbioloxía e Parasitoloxía, Facultade de Veterinaria, Universidade de Santiago de Compostela (USC), Lugo, Spain; 2 Institute for Food Safety and Hygiene, Vetsuisse Faculty, University of Zurich, CH-8057 Zurich, Switzerland

## Abstract

**Background:**

Enteropathogenic *Escherichia coli *(EPEC) and Shigatoxin-producing *Escherichia coli *(STEC) share the ability to introduce attaching-and-effacing (A/E) lesions on intestinal cells. The genetic determinants for the production of A/E lesions are located on the locus of enterocyte effacement (LEE), a pathogenicity island that also contains the genes encoding intimin (*eae*). This study reports information on the occurrence of *eae *positive *E. coli *carried by healthy cattle at the point of slaughter, and on serotypes, intimin variants, and further virulence factors of isolated EPEC and STEC strains.

**Results:**

Of 51 *eae *positive bovine *E. coli *strains, 59% were classified as EPEC and 41% as STEC. EPEC strains belonged to 18 O:H serotypes, six strains to typical EPEC serogroups. EPEC strains harbored a variety of intimin variants with *eae*-β1 being most frequently found. Moreover, nine EPEC strains harbored *ast*A (EAST1), seven *bfpA *(bundlin), and only one strain was positive for the EAF plasmid. We have identified a new intimin gene (η2) in three bovine *bfpA *and *astA*-positive EPEC strains of serotype ONT:H45. STEC strains belonged to seven O:H serotypes with one serotype (O103:H2) accounting for 48% of the strains. The majority of bovine STEC strains (90%) belonged to five serotypes previously reported in association with hemolytic uremic syndrom (HUS), including one O157:H7 STEC strain. STEC strains harbored four intimin variants with *eae*-ε1 and *eae*-γ1 being most frequently found. Moreover, the majority of STEC strains carried only *stx*1 genes (13 strains), and was positive for *ehxA *(18 strains) encoding for Enterohemolysin. Four STEC strains showed a virulence pattern characteristic of highly virulent human strains (*stx*2 and *eae *positive).

**Conclusion:**

Our data confirm that ruminants are an important source of serologically and genetically diverse intimin-harboring *E. coli *strains. Moreover, cattle have not only to be considered as important asymptomatic carriers of O157 STEC but can also be a reservoir of EPEC and *eae *positive non-O157 STEC, which are described in association with human diseases.

## Background

Enteropathogenic *Escherichia coli *(EPEC) and Shiga toxin-producing *Escherichia coli *(STEC) represent two of the at least six different categories of diarrheagenic *E. coli *recognized at present [1]. Unlike other diarrheagenic *E. coli*, EPEC and STEC share the ability to introduce attaching-and-effacing (A/E) lesions on intestinal epithelial cells. A/E lesions are characterized by destruction of the microvillus brush border through restructuring of the underlying cytoskeleton by signal transduction between bacterial and host cells, intimate adherence of strains to the intestinal epithelium, pedestal formation and aggregation of polymerized actin at the sites of bacterial attachment [1,2]. The genetic determinants for the production of A/E lesions are encoded in a pathogenicity island called the locus of enterocyte effacement (LEE) [3]. The LEE encodes the outer membrane protein intimin, which is encoded by the *eae *gene (for *E. coli *attachment effacement) localized in the central region of LEE, a type III secretion system, a number of secreted proteins (ESP), and Tir (for translocated intimin receptor), a protein encoded upstream of the *eae *gene, which is translocated into host cells [2]. Characterization of *eae *genes revealed the existence of different variants. At present, 17 genetic variants (α1, α2, β1, ξR/β2B, δ/κ/β2O, γ1, θ/γ2, ε1, νR/ε2, ζ, η, ι1, μR/ι2, λ, μB, νB, ξB) have been identified (Table 4) [4-9]. The heterogeneous C-terminal (3') end of intimin is responsible for receptor binding, and it is discussed that different intimin variants may be responsible for different host and tissue cell tropism [6,10-12]. Adu-Bobie et al. [4] found that antigenic variation exists within the cell-binding domain of intimins expressed by different clinical human EPEC and STEC isolates and defined four intimin types (α, β, γ, δ) based on type-specific PCR assays that used oligonucleotide primers complementary to the 3'end of specific *eae *genes. Oswald et al. [5] described a type-specific PCR assay which identifies a fifth intimin variant (intimin ε; referred in the present study as ε1). They divided the intimin alleles α, β, and γ based on restriction fragment length polymorphism (RFLP) analysis of PCR products into α1, α2, β1, β2, γ1 and γ2 subtypes. Furthermore, Oswald et al. [5] reclassified the δ type of Adu-Bobie et al. [4] as a β2 subtype (referred in the present study as d/β2O). Tarr and Whitam [13] presented a paper on the molecular evolution of intimin genes in human STEC and EPEC O111 clones and found two new types (ζ and θ). The new ζ intimin type also was identified by Jores et al [11] in bovine STEC and Blanco et al. [7] in ovine STEC. Zhang et al. [6] observed that the sequences of eae-γ2 and eae-θ were almost identical (99%), and they believe that these two sequences should be considered one *eae *variant (γ2/θ), as is referred in the present study. Recently, Zhang et al. [6] determined the sequences of three new intimin variant genes (κ, η, and ι; intimin ι referred in the present study as ι1) found in human STEC strains. The sequence of the 3'variable region of *eae *gene of a new intimin (λ) has been submitted to GenBank by China (unpublished data) (Accession no. AF439538) and the whole intimin λ gene was sequenced by Blanco et al. (unpublished data) from a human strain (Accession no. AJ715409) and Ramachandran et al. [14] (Accession no. AF530557) from a bovine strain. Ramachandran et al. [14] identified three new intimin genes in ruminant *E. coli *strains belonging to serotypes ONT:H- (intimin μ, referred in the present study as μR/ι2), O2-related:H19 (intimin ν, referred in the present study as νR/ε2) and ONT:HNT (intimin ξ, referred in the present study as ξR/β2B) and they have also developed an intimin typing PCR-RFLP scheme that reliably differentiates 14 intimin variants. When Ramachandran et al. [14] submitted to GenBank the nucleotide sequences of μ, ν, and ξ genes these intimin types were designated ι2, ε2 and β2, respectively. Blanco et al. [8] have identified four new intimin variant genes that they originally designated as β2, μ, ν, and ξ when the sequences were submitted to EMBL Nucleotide Sequence Database, and before knowing the results obtained by Ramachandran et al. [14]. The intimin β2 found by Blanco et al. (unpublished data) in human typical EPEC strains of classical EPEC serotype O119:H6 is identical to intimin ξ described by Ramachandran et al. [14] in one bovine strain of serotype ONT:HNT. Thus, in this study our β2 intimin is referred as ξR/β2B. The other three intimins (μ, ν, and ξ) discovered by Blanco et al. [[8], unpublished data] are different to the existing intimin types and are referred to as μB, νB, and ξB in this study, respectively.

EPEC strains are defined as *eae*-haboring diarrheagenic *E. coli *that possess the ability to form A/E lesions on intestinal cells and that do not possess Shiga toxin genes [15]. Most EPEC strains belong to a series of O antigenic groups known as EPEC serogroups: O26, O55, O86, O111, O114, O119, O125, O126, O127, O128, O142, and O158. EPEC are further classified as typical, when possessing the EAF (for EPEC adherence factor) plasmid that encodes localized adherence (LA) on cultured epithelial cells mediated by the Bundle Forming Pilus (BFP); whereas atypical EPEC strains do not possess the EAF plasmid [15,16]. Typical EPEC, a major cause of infant diarrhea in developing countries, are rare in industrialized countries, where atypical EPEC seem to be a more important cause of diarrhea [1,16]. Typical and atypical EPEC strains usually belong to certain serotype clusters, differ in their adherence patterns on cultured epithelial cells (typical: LA; atypical: diffuse adherence (DA); aggregative adherence (AA); localized adherence-like adherence (LAL)), are typically found in different hosts (typical EPEC strains have only been recovered from humans), and display differences in their intimin variants [16]. Atypical EPEC appear to be more closely related to STEC and as such are considered emerging pathogens [16,17].

STEC are responsible for a number of human gastrointestinal diseases, including diarrhea, bloody diarrhea and hemorrhagic colitis (HC). In a proportion of individuals, particularly in children, these conditions may be complicated by neurological and renal sequelae, including hemolytic-uremic syndrome (HUS) [18-20]. Most outbreaks and sporadic cases of HC and HUS have been attributed to O157:H7 STEC strains [20-22]. In contrast, especially in continental Europe, infections by non-O157 STEC, such as O26:H11/H-, O91:H21/H-, O103:H2, O111:H-, O113:H21, O117:H7, O118:H16, O121:H19, O128:H2/H-, O145:H28/H-, and O146:H21, are more common and frequently associated with severe illness in humans [1,23-27]. Probably, non-O157 STEC also play a more important role in disease in Argentina [28], Australia [29], Chile [1], and South Africa [1]. STEC are characterized by elaborating two groups of potent phage-encoded cytotoxins called Shiga toxins [18,19]. Pathogenic STEC very often possess other putative virulence factors as intimin (*eae*) and Enterohemolysin (*ehxA*) [19,30,31].

Although a number of studies have determined the *eae *subtypes of STEC strains isolated from humans, only a limited number of studies have been undertaken to determine the intimin variants from a diverse collection of STEC and EPEC isolated from cattle. Ruminants, especially cattle, represent an important reservoir of atypical EPEC and STEC [16,20,32,33]. This study reports information on the occurrence of *eae *positive *E. coli *carried by healthy cattle at the point of slaughter, and on serotypes, intimin variants, and further virulence factors of isolated EPEC and STEC strains.

## Results

### Detection and isolation of *eae *positive *E. coli *strains

Of the 330 fecal samples collected from cattle at slaughter, 132 (40%) tested positive for *eae *genes. From 51 randomly selected *eae *PCR-positive samples, 51 *eae *positive *E. coli *strains were isolated by colony dot blot hybridization, confirmed as *E. coli *by biochemical properties, and further characterized by phenotypic and genotypic traits. Thirty strains carried no *stx *genes and were therefore classified as EPEC, whereas the remaining 21 strains possessed at least one Shiga toxin gene, and were therefore considered as STEC.

### Serotypes of bovine *eae *positive *E. coli *strains

The 51 *eae *positive *E. coli *strains belonged to 18 O serogroups, 9 H types, and 23 O:H serotypes. However, 46% of strains were of three serogroups, namely O26 (6 strains), O103 (12 strains), and O145 (5 strains), and 43% belonged to two H types, namely H2 (17 strains), and H45 (5 strains). Thirty-two percent of strains were of only three serotypes: O26:H- (3 strains), O103:H2 (11 strains), and O145:H- (5 strains). EPEC strains belonged to 18 O:H serotypes and eight strains were nontypeable (Table 1). STEC strains comprised seven O:H serotypes with one serotype (O103:H2) accounting for 48% of STEC strains (Table 2). Furthermore, all serotypes found in this study have been previously reported in human STEC. Five (O5:H-, O26:H-, O103:H2, O145:H-, and O157:H7) of the STEC serotypes comprising 19 (90%) strains have previously been associated with human STEC causing HUS. Serotypes O26:H- and O103:H2 were found in both EPEC and STEC strains.

### Typing of intimin (*eae*) genes

Overall, the 51 isolated *eae *positive bovine *E. coli *strains comprised a variety of 10 intimin variants, namely α1(1 strain), β1 (17 strains), ξR/β2B (2 strains), γ1 (6 strains), γ2/θ (2 strains), ε1 (13 strains), ζ (3 strains), η2 (3 strains), ι1 (2 strains), and κ (2 strains). Intimins α2, δ/β2O, γ1, θ/γ2, ν R/ε2, η1, μR/ι2, λ, μB, νB, and ξB were neither detected in bovine EPEC nor in bovine STEC. EPEC strains harbored a variety of nine intimin variants (α1, β1, ξR/β2B, κ, θ/γ2, ε1, ζ, η2, ι1). In 15 strains (52%) *eae*-β (twelve times β1, twice β2), which was associated with nine serotypes, in three strains *eae*-ε1, in three strains *eae*-ζ, in three strains *eae*-η2, in two strains *eae*-ι1, in two strains *eae*-κ, in one strain *eae*-α1, and in one strain *eae*-γ2/θ were detected (Table 1). In contrast, STEC strains harbored only four intimin variants (β1,γ1,γ2/θ, ε1) with *eae*-ε1 (10 strains, all of serotype O103:H2) and *eae*-γ1 (5 strains of serotype O145:H-, and one strain of serotype O157:H7) being most frequently found (Table 2). Intimins β1, ε1 and γ2/θ were present in both EPEC and STEC strains.

### Identification of a new intimin variant gene (eae-η2). Sequence comparison

The complete nucleotide sequence of the new η2 variant gene in three bovine *bfpA *and *astA*-positive EPEC strains of serotype ONT:H45 was determined. Furthermore, a fragment (566 bp strain FV5126 to 1728 bp strain FV5114/2) of the 3'variable region of the *eae *gene of seven representative strains was amplified and sequenced. The *eae *sequences were deposited in the European Bioinformatics Institute (EMBL Nucleotide Sequence Database) and the accession numbers assigned are indicated in table 3.

By using CLUSTAL W for optimal sequence alignment, we determined the genetic relationship of the new intimin gene (η2) and the remaining *eae *variants (Table 4). Thus, the *eae*-η2 sequence is very similar to *eae*-η1 (identy of 98%). Phylogenetic analysis revealed six groups of the closely related intimin genes: (i) α1, α2, ζ, and νB; (ii) β1, δ/β2O, κ, ξR/β2B; (iii) ε1, ξB, η1, η2 and νR/ε2; (iv) γ1, μB, and γ2/θ; (v) λ ; and (vi) ι1 and μR/ι2.

### Further characterization of EPEC and STEC strains

Nine EPEC strains harbored the *ast*A gene encoding for EAST1 (Table 1). Additionally, three *ast*A positive strains of serotype ONT:H45 harbored the *bfpA *gene encoding bundlin, whereas only one strain was positive for the EAF plasmid. Overall, 23 different associations of serotypes with virulence factors were identified in EPEC strains.

Of the 21 *eae *positive *E. coli *harboring genes encoding for Shiga toxins, the majority (13 strains) carried only *stx*1 genes, 4 strains only *stx*2 genes, and 4 strains both toxin genes (Table 2). The only *stx*1 positive strains belonged to serotypes O5:H- (2 strains), O103:H2 (10 strains) and O111:H21 (1 strain), the only *stx*2 positive strains to serotypes O26:H-, O26:H2, O145:H-, and O157:H7, and all the strains positive for both toxin genes to serotype O145:H-. Moreover, the majority of STEC strains (86%) tested positive for the *ehxA *gene encoding for Enterohemolysin. Overall, ten different associations of serotypes with virulence factors were identified.

## Discussion

Of 51 *eae *positive *E. coli *strains, 59% were classified as EPEC and 41% as STEC. The serogroups O26, O103, and O145 and serotypes O26:H-, O103:H2, O145:H- were most frequently found. Comparable distributions of EPEC (57%) and STEC (43%) strains and serogroups (44% of strains serotyped as O26, O103, O145, or O156) were reported in strains isolated from healthy cattle in Japan [34]. Of the typical EPEC serogroups (O26, O55, O86, O111, O114, O119, O125, O126, O127, O128, O142, and O156), only six strains were found in the present study: four O26 strains, one O119 strain, and one O128 strain. In bovine STEC strains, only a restricted number of serotypes have been most commonly found [8,20]: the predominant serotype in the majority of surveys realized in Europe was O113:H21, in America and Australia O26:H11, and in Japan O45:H8/H- and O145:H-. As in the present study, authors in Argentina, Canada, France, Germany, Spain, and the United States have found that many STEC recovered from cattle belonged to serotypes previously associated with human disease [8,35-38].

Intimin mediates the intimate bacterial attachment to the host cell surface of EPEC and STEC, and is required for the formation of the characteristic A/E lesions. In EPEC and STEC strains isolated from human patients and from healthy infants from Germany and Australia, intimin variants α1, β1, γ1 and γ2/θ were most frequently detected [5,6,8,17,26]. Moreover, STEC serotypes commonly recovered from outbreaks of HUS and hemorrhagic colitis (O157:H7/H-; O111:H-; O26:H11/H-) typically possess intimin γ1, γ2/θ, and β1 [5,6,8,14].

Overall, the 51 isolated *eae *positive bovine *E. coli *strains comprised a variety of 10 intimin variants, and most of them harbored *eae*-β1(33%), *eae*-ε1 (26%) and *eae*-γ1(16%). As it is known that different intimin variants are associated with distinctive phylogenetic lineages of LEE-positive *E. coli *[4,39,40], the identified variety of bovine *eae *positive strains further substantiates the reservoir function of cattle for infections of humans with LEE positive strains.

Intimin β1 appears to be the most widespread variant [7,8,14,27,39,41,42]. In that it has been found in both EPEC and STEC strains from humans and several animal species, and its presence is associated with multiple *E. coli *serotypes. Strains harboring *eae*-β1 include important human diarrheagenic serotypes such as EPEC O26:H11, O111:H2, O114:H2, O126:H2, O128:H2 and STEC O26:H11/H-, O118:H16 and O177:H- [5,26,27], serotypes partly also detected in this study.

Intimin ε1 was described for the first time in human and bovine STEC of serogroup O8, O11, O45, O103, O121, and O165 [5]. In this study, *eae*-ε1, was detected in three bovine EPEC strains including one O103:H2 strain, and in all 9 bovine STEC O103:H2 strains. Furthermore, in a recent study performed on sheep in Switzerland, intimin ε1 was detected in all STEC O103:H2 [43]. STEC O103:H2 strains have frequently been associated with HUS and hemorrhagic colitis [26,27,44,45].

Intimin γ1 was restricted to STEC and associated with serotypes considered as highly pathogenic to humans: O145:H- (five strains) and, O157:H7 (one strain). In addition to these serotypes, *eae*-γ1 was previously described in association with STEC O145:H28, and with EPEC O55:H7/H-, from which STEC O157:H7 are believed to have evolved [8,26,27,46].

Interestingly, one bovine EPEC O157:H45 strain harboring *eae*-α1 was detected. Moreover, *eae*-α1 was also recently detected in bovine EPEC O157:H45 [33], in two EPEC O142:H6 isolated from monkeys [47] and in a human STEC O177:H7 strain [25]. These findings disagree with the suggested restriction of intimin α1 to human EPEC strains of serotypes O55:H6, O127:H6, H34/H-, O142:H6 [4,5].

We have identified a new intimin gene (η2) in three bovine *bfpA *and *astA*-positive EPEC strains of serotype ONT:H45. The complete nucleotide sequences of the new eae-η2 (AJ879898, AJ879899, AJ879900) variant genes of the three strains were determined. The new intimin η2 also was found in one EPEC ONT:H45 strain isolated from a patient with diarrhea in Spain by Blanco et al. (unpublished data, EMBL nucleotide sequence AJ876652). Recently, a food-borne outbreak of diarrhea involving 41 students (ages 12 to 15) was reported in Japan by Yatsuyanagi et al. [48]. The implicated organism was a EPEC ONT:H45, which hybridized with the probes for *eaeA*, *astA *and *bfpA *genes.

In our study, about one third of EPEC strains harbored *ast*A as further putative virulence factor; whereas only seven EPEC strains harbored *bfpA *encoding bundling, the structural subunit of the bundle-forming pilus in typical EPEC strains, and only one EPEC of serotype O157:H45 was positive for the EAF plasmid. Overall, *bfpA *seems to be isolated very rarely from bovine EPEC strains, since there are scarcely any reports of *E. coli *strains with *bfpA *isolated from cattle. However, in a recent study, we characterized 11 O157:H45 EPEC strains isolated from cattle in Switzerland and found 10 *bfpA *positive strains among them [33].

To assess the pathogenicity of STEC, further evaluation of virulence factors in addition to serotype is necessary. O157 and non-O157 STEC strains isolated from patients with severe symptoms such as bloody diarrhea, HC, and HUS frequently show a typical virulence spectrum, with such strains tending to be *stx*2 and *eae *positive [49-51]. Moreover, it was previously shown that bovine and human strains harboring the *eae *gene were statistically more likely to be positive for the *ehxA *gene encoding for Enterohemolysin [7,26,52]. However, the impact of Enterohemolysin, which was found in the majority of STEC strains, is controversially discussed. Additionally, STEC commonly recovered from outbreaks of HUS and HC typically possessed *eae*-β1, *eae*-γ1 and γ2/θ [5-7,39]. In consequence, apart of the *stx*2 gene, which was previously reported to correlate with severe disease in humans, intimin subtyping may facilitate further understanding of associations among serotype, *eae *and *stx *subtype. In consequence, seven STEC strains (14% of all *eae *positive strains and 33% of STEC strains) showed a virulence pattern characteristic of highly virulent human strains: four O145:H- strains harboring *stx*1, *stx*2, *eae*-γ1, and *ehxA*, one O26:H- strain harboring *stx*2, *eae*-β1, and *ehxA*, one O145:H- strain harboring *stx*2, *eae*-γ1, and *ehxA*, and one O157:H7 strain harboring *stx*2, *eae*-γ1, and *ehxA*.

## Conclusion

Our data confirm that ruminants are an important source of serologically and genetically diverse intimin-harboring *E. coli *strains. Moreover, cattle have not only to be considered as important asymptomatic carriers of O157 STEC but can also be a reservoir of EPEC and *eae *positive non-O157 STEC, which are described in association with human diseases. The fecal carriage of foodborne pathogens among livestock animals at slaughter is strongly correlated with the hazard of carcasses contamination. In order to reduce the risk represented by STEC and EPEC, the maintenance of slaughter hygiene is consequently of central importance in meat production.

## Methods

### *E. coli *strains

From 330 fecal samples, collected from Swiss cattle at slaughter, 10 g were each enriched in 100 ml brilliant green bile broth (BBL, Cockeysville, Md.) at 37°C for 24 h. The enriched samples were streaked onto sheep blood agar (Difco Laboratories, Detroit, Mich.; 5% sheep blood Oxoid, Hampshire, UK), and after incubation at 37°C for another 24 h, the colonies were washed off with 2 ml of 0.85% saline solution. Two μl of each plate eluate were then evaluated by PCR with primers EAE-1 and EAE-2 (Table 5) targeting sequences at the 5' *eae *conserved region detecting all types of *eae *described at the moment. From the 132 *eae *PCR-positive samples, 51 *eae *PCR-positive samples were then randomly selected for strain isolation with an *eae *DNA probe and colony dot-blot hybridization.

The *eae *probes were prepared by labeling *eae*-PCR amplicons from *E. coli *O157:H7 strain 857/03 with DIG High Prime kit (Roche, Mannheim, Germany). Briefly, for colony hybridization, the 51 *eae *positive samples were plated onto sheep blood agar and incubated overnight at 37°C. Colonies were transferred to a nylon membrane (Roche), and lysed following standard methods. After washing, crosslinking, and prehybridization in DIG-Easy-Hyb buffer (Roche) at 37°C for about 30 min, hybridization of membranes with *eae *DNA probes was performed overnight at 42°C. After washing in pre-heated primary and secondary wash buffers, the presence of labeled probe was detected with in alkaline phosphatase-conjugated antibody detection kit and NBT/BCIP stock solution according to the instructions of the manufacturer (Roche). Positive colonies were picked from the original sheep blood agar and confirmed as *E. coli *by biochemical properties (acid production from mannitol, the o-nitrophenyl-β-D-galactopyranoside (ONPG) test, H_2_S and indole production, and proof of urease and lysine decarboxylase activities) and by PCR to be *eae *positive [7]. One randomly chosen colony per sample was used for further strain characterization.

### Further strain characterization

Determination of O and H antigens was performed by the method described by Guinée et al. [53] with all available O (O1 to O185) and H (H1 to H56) antisera. *E. coli *isolates that are nonmotile are described as having an H- flagellum type. Antisera were obtained and absorbed with corresponding cross-reaction antigens to remove nonspecific agglutinins. O antisera were produced in the Laboratorio de Referencia *E. coli *(LREC) , and H antisera were obtained from the Statens Serum Institut (Copenhagen, Denmark). *E. coli *strains representing the new O groups O182 to O185 [unpublished data] were kindly provided by Flemming Scheutz (International Escherichia Centre, Statens Serum Institut, Copenhagen, Denmark).

All PCR assays applied in this study for characterization of *eae *positive *E. coli *strains were performed in a T3 thermocycler (Biometra, Göttingen, Germany). PCR reagents were purchased from PROMEGA (Madison, Wis.), and primers were synthesized by MICROSYNTH (Balgach, Switzerland). The 50-μl PCR mixtures normally consisted of 2 μl of bacterial suspension boiled at 100°C for 5 min in 42 μl of double-distilled water, 5 μl of 10-fold-concentrated polymerase synthesis buffer containing 2.0 mM MgCl_2_, 200 μM (each) desoxynucleosid triphosphate (dNTP), 30 pmol of each primer, and 2.5 U of *Taq *DNA polymerase. The PCR primers, target sequences, product sizes and references are listed in Tables 5 and 6. Differences in the normal PCR mixture and cycling conditions for each PCR were previously described in the cited literature. To identify *eae *variants, intimin type specific PCR assays using primers complementary to the heterogeneous 3' end of intimin genes were performed [8,9]. For detection of further putative virulence genes all strains were examined for the presence of *stx *genes with primers VT1 and VT2 [54]. *Stx *positive strains were examined for the presence of *stx*1 and *stx*2 genes [55,56]. *Stx *negative strains were further examined for the presence of the EAF plasmid [57], the *bfpA *gene on the EAF plasmid [58], and the *astA *gene encoding EAST1 [59].

### Sequencing of the intimin (*eae*) genes

The nucleotide sequence of the amplification products purified with a QIAquick DNA purification kit (Qiagen) was determined by the dideoxynucleotide triphosphate chain termination method of Sanger, with the BigDye Terminator v3.1 Cycle Sequencing Kit and an ABI 3100 Genetic Analyzer (Applied Bio-Systems).

### Phylogenetic analyses

Genetic distances and phylogenetic trees of *eae *sequences were calculated and constructed with the CLUSTAL W program [60]included in the EMBL sofware .

### Nucleotide sequence accession numbers

The *eae *sequences of strains analyzed were deposited in the European Bioinformatics Institute (EMBL Nucleotide Sequence Database) and the accession numbers assigned are indicated in Table 3.

## Authors' contributions

RS and JB designed the study and drafted the manuscript. SS and TT isolated the strains, JEB has done serotyping of the strains, MB designed the primers used for PCR typing of intimins, performed the PCR typing of *eae *genes and sequenced the *eae *genes, GD also participated in the PCR typing of intimins, and CZ was responsible for further strain characterization. All authors read, commented on and approved of the final manuscript.

## Figures and Tables

**Table 1:** Serotypes and virulence factors of EPEC strains isolated from cattle in Switzerland (n = 30)

Serotype	No. of strains	*eae*	*astA*	*bfpA*
O2:H45	1	κ	-	-
O8:H19	1	β1	+	-
O10:H-	1	γ2/θ	-	-
O15:H2	3	β1	-	-
O26:H-	1	β1	+	-
O26:H-	1	β1	-	-
O26:H11	2	β1	-	-
035:H2	1	β1	-	-
O64:H25	1	β1	-	-
O77:H19	1	β1	-	-
O103:H2	1	ε1	-	-
O103:H8	1	ι1	+	+
O113:H6	2	ξR/β2B	-	-
O119:H8	1	ι1	-	+
O128:H2	1	β1	-	-
O156:H25	1	ζ	-	-
O157:H45	1	α1	+	+
O177:H11	1	β1	+	+
ONT:H-	1	ε1	-	-
ONT:H-	1	κ	-	-
ONT:H19	1	ε1	+	-
ONT:H25	2	ζ	-	-
ONT:H45	3	η2	+	+

ONT: O antigen nontypeable with O1 to O185 antisera.

**Table 2:** Serotypes and virulence factors of *eae *positive STEC strains isolated from cattle in Switzerland (n = 21)

Serotype	No. of strains	*stx*	*eae*	*ehxA*
O5:H- ^b^	1	*stx*1	β1	+
O5:H- ^b^	1	*stx*1	β1	-
O26:H- ^b^	1	*stx2*	β1	+
O26:H2 ^a,c^	1	*stx2*	β1	-
**O103:H2 **^b^	9	*stx*1	ε1	+
**O103:H2 **^b^	1	*stx*1	ε1	-
O111:H21 ^a^	1	*stx*1	γ2/θ	+
**O145:H- **^b^	4	*stx*1, *stx*2	γ1	+
**O145:H- **^b^	1	*stx2*	γ1	+
**O157:H7 **^b^	1	*stx*2	γ1	+

^a ^Serotypes previously found as human STEC strains

^b ^Serotypes previously associated with human STEC strains that caused HUS

^c ^Serotypes not yet reported within bovine STEC strains

Serotypes that caused human outbreaks are in **bold**

**Table 3:** Accession numbers of sequenced *eae *genes of some EPEC strains

Strain	Origin	Serotype	Intimin	EMBL nucleotide sequence	*bfpA*	*EAF plasmid*	*stx1 stx2*
FV5109-4113/1	healthy cattle^1^	ONT:H45	η2	AJ879898	+	-	-
FV5113-4115/2	healthy cattle^1^	ONT:H45	η2	AJ879899	+	-	-
FV5114/1-3933/51	healthy cattle^1^	ONT:H45	η2	AJ879900	+	-	-
H03-53199a	patient with diarrhea^2^	ONT:H45	η2	AJ876652	nd	nd	-
FV5114/2-3933/52	healthy cattle^1^	O156:H25	ζ	AJ879901	-	-	-
FV5125-4125/1	healthy cattle^1^	O157:H45	α1	AJ879902	+	-	-
FV5123-4286/1	healthy cattle^1^	O2:H45	κ	AJ879903	-	-	-
FV5133-3988/1	healthy cattle^1^	ONT:H-	κ	AJ879904	-	-	-
FV5126-3951/1	healthy cattle^1^	O119:H8	ι1	AJ879905	+	-	-
FV5120-4075/1	healthy cattle^1^	O103:H8	ι1	AJ879906	+	-	-
FV3854-1070/1	healthy cattle^3^	O157:H45	α1	AJ879907	+	-	-

^1^from Switzerland; ^2^from Spain; ^3^Stephan et al. [33]; nd: not done

**Table 4:** Genetic relationship of the new intimin gene (η2) (AJ879898, AJ879899, AJ879900) detected in bovine EPEC strains of serotype ONT:H45 and the remaining *eae *variants: Pairwise alignments calculated with CLUSTAL W

Designation of intimins in this study	Designation of intimins in previous studies (References)	ORF length (bp)	Reference strain	Serotype	Origin	Accession number	Genetic relationship identities
α1	α (4) and α1 (5)	2820	E2348/69	O127:H6	Human	M58154	87%
α2	α (4) and α2 (5)	2820	*E. coli*	O125:H6	Human	AF530555	87%
β1	β (4) and β1 (8)	2820	RDEC1	O15:H-	Rabbit	AF200363	84%
ξR/β2B	ξ (14) and β2 (8)	2820	KB411	ONT:HNT	Bovine	AF530556	88%
ξR/β2B	ξ (14) and β2 (8)	2820	FV359	O119:H6	Human	AJ715407	88%
δ/β2O	δ (4) and β2 (5)	2820	BL152.1	O86:H34	Human	AJ875027	87%
κ	κ (6)	2820	6044/95	O118:H5	Human	AJ308552	87%
γ1	γ (4) and γ1(5)	2805	EDL933	O157:H7	Human	AF071034	86%
γ2/θ	γ2 (5) and θ (13)	2808	CL-37	O111:H8	Human	AF449418	84%
ε1	ε(5)	2847	PMK5	O103:H2	Human	AF116899	92%
νR/ε2	ν (14) and ε2 (GenBank AF530554)	2847	VR64/4	O2related:H19	Ovine	AF530554	94%
ζ	ζ (13)	2817	4795/95	O84:H4	Human	AJ271407	86%
η1	η (6)	2847	CF11201	O125:H-	Human	AJ308550	98%
η2	new (this study)	2847	H03/53199a	ONT:H45	Human	AJ876652	100%
ι1	ι (6)	2814	7476/96	O145:H4	Human	AJ308551	88%
μR/ι2	μ (14) and ι2 (GenBank AF530553)	2814	VR45	OR:H-	Ovine	AF530553	83%
λ	λ (8, 14)	2817	EPEC-68.4	O34:H-	Human	AJ715409	86%
μB	μ (8)	2808	EPEC-373	O55:H51	Human	AJ705049	87%
νB	ν (8)	2823	IH1229a	O10:H-	Human	AJ705050	82%
ξB	ξ (8)	2847	STEC-B49	O80:H-	Bovine	AJ705051	91%

**Table 5:** Sequences of oligonucleotide primers used for typing of *eae *intimin gene

Gene	Primer	Oligonucleotide sequence (5'-3')^c^	Fragment size (bp)	Annealing temperature	Primer coordinates	Accession number	Reference
*eae*^a^	EAE-1 EAE-2	GGAACGGCAGAGGTTAATCTGCAG GGCGCTCATCATAGTCTTTC	775	55°C	1441–1460 2193–2215	AF022236	7
*eae*-α1	EAE-FB EAE-A	AAAACCGCGGAGATGACTTC CACTCTTCGCATCTTGAGCT	820	60°C	1909–1928 2709–2728	AF022236	8
*eae*-α2	IH2498aF IH2498aR	AGACCTTAGGTACATTAAGTAAGC TCCTGAGAAGAGGGTAATC	517	60°C	2099–2122 2597–2615	AF530555	8
*eae*-β1	B1A B1B	ACTTCGCCACTTAATGCCAGC TTGCAGCACCCCATGTTGAAT	730	66°C	1924–1944 2633–2653	AF453441	9
*eae*-ξR/β2B^b,e^	B2A B2B	AAGGGGGGAACCCCTGTGTCA ATTTATTCGCAGCCCCCCACG	604	66°C	2056–2076 2639–2659	AF530556 AJ715407	9
*eae*-δ/κ/β2O^c^	EAE-FB EAE-D	AAAACCGCGGAGATGACTTC CTTGATACACCCGATGGTAAC	833	60°C	1909–1928 2721–2741	U66102	8
*eae*-γ1	EAE-FB EAE-C1	AAAACCGCGGAGATGACTTC AGAACGCTGCTCACTAGATGTC	804	60°C	1909–1928 2691–2712	AF071034	8
*eae*-θ/γ2^d^	EAE-FB EAE-C2	AAAACCGCGGAGATGACTTC CTGATATTTTATCAGCTTCA	808	58°C	1909–1928 2697–2716	AF025311	8
*eae*-ε1	EAE-FB LP5	AAAACCGCGGAGATGACTTC AGCTCACTCGTAGATGACGGCAAGCG	722	66°C	1909–1928 2605–2630	AF116899	8
*eae*-νR/ε2^e^	EAE-E2F EAE-E2R	AATACAGAAGTTAAGGCAT ACGACCACTATTCATTTC	378	58°C	2230–2248 2590–2607	AF530554	9
*eae*-ζ	Z1 Z2	GGTAAGCCGTTATCTGCC ATAGCAAGTGGGGTGAAG	206	62°C	2062–2079 2250–2267	AF449417	8
*eae*-η	EAE-FB ETA-B	AAAACCGCGGAGATGACTTC TAAGCGACCACTATTCGTG	702	60°C	1899–1918 2582–2600	AJ308550	9
*eae*-ι1	EAE-FB IOTA-B	AAAACCGCGGAGATGACTTC GTCATATTTAACTTTTACACTA	651	55°C	1909–1928 2538–2559	AJ308551	9
*eae*-μR/ι2^e^	Iota2-F Iota2-R	CTGGTAAAGCGATAGTCAAAC GCGTTTTTGAAGAAACATTTTGC	936	58°C	1850–1870 2763–2785	AF530553	9
*eae*-λ	68.4F 68.4R	CGGTCAGCCTGTGAAGGGC ATAGATGCCTCTTCCGGTATT	466	64°C	2061–2079 2506–2526	AJ715409	8
*eae*-μB^f^	EAE-FB FV373R	AAAACCGCGGAGATGACTTC ACTCATCATAATAAGCTTTTTGG	665	60°C	1909–1928 2541–2563	AJ705049	9
*eae*-νB^f^	IH1229aF IH1229aR	CACAGCTTACAATTGATAACA CTCACTATAAGTCATACGACT	311	60°C	269–289 559–579	AJ705050	8
*eae*-ξB^f^	EAE-FB B49R	AAAACCGCGGAGATGACTTC ACCACCTTTAGCAGTCAATTTG	468	66°C	1909–1928 363–384	AF116899 AJ705051	8

^a^Universal oligonucleotide primer pair EAE1 and EAE-2 wih homology to the 5'conserved region of *eae *gene (detects all types of *eae *variants described at the moment). Primers used to detect the eae gene.

^b^The intimin β2 of Blanco et al. (8) is identical to intimin ξ described by Ramachandran et al. (14). In this study our β2 is referred as ξR/β2B.

^c^The intimin δ of Adu-Bobie et al. (4) is identical to intimin κ of Zhan et al. (6). The intimin δ of Adu-Bobie et al. (4) was also termed β2 by Oswald et al. (5). Thus, in the present study this intimin is referred as δ/κ/β2O.

^d^The intimin θ of Tarr and Whittam (13) is the same as intimin γ2 of Oswald et al. (5).

^e^Intimins μ, ν, and ξ described by Ramachandran et al. (14) in ruminant *E. coli *strains. When Ramachandran et al. (14) submitted the nucleotide sequences of μ, ν, and ξ genes these intimin types were designated ι2, ε2 and β2, respectively.

^f^Intimins μ, ν, and ξ described by Blanco et al. (8, unpublished results).

**Table 6:** Sequences of oligonucleotide primers used for detection other virulence genes

Target	Primer	Oligonucleotide sequence (5'-3') Sequence	Product size	Reference
*stx*	VT1 VT2	ATTGAGCAAAATAATTTATAT GTG TGATGATGGCAATTCAGTAT	523 bp 520 bp	54
*stx*1	KS7 KS8	CCCGGATCCATGAAAAAAACATTATTAATAGC CCCGAATTCAGCTATTCTGAGTCAACG	285 bp	55
*stx*2	VT2-e VT2-f	AATACATTATGGGAAAGTAATA TAAACTGCACTTCAGCAAAT	348 bp	56
*ehxA*	HlyA1 HlyA2	GGTGCAGCAGAAAAAGTTGTAG TCTCGCCTGATAGTGTTTGGTA	311 bp	31
*astA*	EAST11a EAST11b	CCATCAACACAGTATATCCGA GGTCGCGAGTGACGGCTTTGT	111 bp	59
EAF	EAF1 EAF25	CAGGGTAAAAGAAAGATGATAA TATGGGGACCATGTATTATCA	397 bp	57
*bfpA*	EP1 EP2	AATGGTGCTTGCGCTTGCTGC GCCGCTTTATCCAACCTGGTA	326 bp	58
